# Physicians and War

**DOI:** 10.1016/j.shj.2022.100028

**Published:** 2022-04-26

**Authors:** Anthony N. DeMaria

**Affiliations:** Judy and Jack White Chair in Cardiology, Sulpizio Cardiovascular Center, University of California, San Diego, La Jolla, California, USA

On the most elementary level, the concept of war is totally insane. The thought that cogent beings cannot resolve an issue in any other way than killing each other challenges the concept that they are actually rational. While clearly one may have no choice but to defend themselves if attacked, that does not detract from the fundamental lunacy of the act of killing. Nevertheless, we now again are witnessing a full-scale war in Europe, and again it is inconceivable that any adequate justification exists for the initiation of the conflict. The result is that many young men and women will die, many others will be injured, and even more will have their quality of life diminished or destroyed.

While the concept of war is, or should be, repulsive to everyone, it has a particular effect upon physicians. After all, we dedicate our lives to reduce mortality and relieve suffering and have made considerable sacrifices to be able to do so. We sometimes make heroic efforts to prolong survival, not infrequently for elderly individuals who are near the end of life and have many comorbidities. This is certainly common in the field of structural heart disease. To then see young, healthy individuals in the prime of life killed or maimed is particularly unsettling and depressing.

I was involved in the care of young, otherwise-healthy individuals who had sustained serious injuries or infections in the Viet Nam War. Helping to take care of these individuals stirred a variety of emotions and thoughts. I was impressed, of course, with their bravery and almost universal acceptance of their injuries with no or little resentment. My initial reaction, however, was to cry out that we have to stop this insane war. I had to wonder if my goals of saving lives and reducing morbidity could not be better achieved in politics than medicine. It also seemed that my dedication to those goals was even amplified in attending to the wounded.

A unique consideration in terms of physicians and war, and one I was not confronted with, was our role in caring for the enemy. We take an oath to care for the sick and injured without regard for any status. I saw a news program that interviewed a Ukrainian doctor and expressed surprise that he was caring for wounded Russian soldiers. I think that caring for the enemy is a particularly important role that we can fulfill. It testifies to our basic value for life and respect for humanity and sets an example that we are all human beings so that killing each other is irrational. On a basic level, the desire for peace and a healthy and happy family is nearly universal. As the cliché goes, the people who decide to conduct war rarely go to the battle themselves.

An additional aspect of the medical profession and war involves the creation of weapons of mass, or even individual, destruction. Should physicians have any role in biologic or chemical warfare? It seems completely paradoxical to devote energy to research in preventing or curing disease on the one hand and then working on methods to produce disease on the other. Clearly, it is necessary to research and prepare for the possibility that others might use such destructive weapons. However, the thought of generating such tools of mass and impersonal destruction that would be actively used is abhorrent. Just as sins by religious and crimes by police are especially appalling, so are weapons to kill or injure that are created by doctors.

As I write this, we are living in a time when all media are full of scenes of death, injury, and destruction. Even worse, the victims are often civilians. It is impossible for anyone to view these images without a profound sense of sadness. However, in a certain sense I think these scenes are even more difficult and impactful for physicians. We see videos of bombs exploding near children while we prepare to place a transcatheter aortic valve in an octogenarian aortic stenosis patient with coronary disease and chronic obstructive pulmonary disease. While we compartmentalize the war from what we do every day, it is almost impossible to fully reconcile these 2 activities. We can be consoled by the fact that our efforts to benefit society offset to some degree the actions of others to degrade it. Moreover, we can feel ennobled by the heroic efforts of our physician colleagues in rendering care not only to their besieged countrymen but also to the enemy. We can prepare for the day when this insane war will end and advocate as vigorously as possible that another war never be allowed to occur.

## Funding

The author has no funding to report.

## Disclosure statement

The author reports no conflict of interest.

